# COVID-19 Pneumonia Diagnosed by Bronchoalveolar Lavage Fluid, With CD4 T-cell Depletion Contributing to Prolonged Infection: Two Case Reports

**DOI:** 10.7759/cureus.74380

**Published:** 2024-11-25

**Authors:** Saya Tsukida, Masato Hongo, Kyota Akasaki, Takashi Sone, Koichi Nishi

**Affiliations:** 1 Internal Medicine, Keiju Medical Center, Nanao, JPN; 2 Internal Medicine, Fukui-ken Saiseikai Hospital, Fukui, JPN; 3 Internal Medicine, Ishikawa Prefectural Central Hospital, Kanazawa, JPN

**Keywords:** anti-cd20 therapy, bendamustine, bronchoalveolar lavage fluid, cd4+ t-lymphocytopenia, covid-19, nasopharyngeal swab, sars-cov-2 pcr

## Abstract

Irrespective of the underlying disease, patients treated with cluster of differentiation 20 (CD20) antibodies have a higher risk of severe acute respiratory syndrome coronavirus 2 (SARS-CoV-2) long or severe infection, and there are pitfalls in this diagnosis. We herein report two patients with COVID-19 pneumonia diagnosed by bronchoalveolar lavage fluid (BALF) during lymphoma remission. Nasopharyngeal swabs (NSs) were polymerase chain reaction (PCR)-negative for SARS-CoV-2, and the virus was only detectable in the lungs. In patients with B-cell depletion, the early performance of bronchoalveolar lavage (BAL) is important for diagnosing COVID-19 pneumonia and ruling out opportunistic infections when any evidence of suspected viral pneumonia is observed on computed tomography (CT), even if the NS specimens are PCR-negative and they have no upper respiratory symptoms. In addition, blood tests with lymphocytopenia, BALF with decreased CD4/CD8 ratio, and increased neutralizing antibody titer suggested that not only low humoral immune responses but also CD4 T-cell depletion by bendamustine were associated with virus clearance. Even if neutralizing antibodies are adequate, we must be careful of prolonged COVID-19 due to CD4 T-cell depletion and low humoral immune responses.

## Introduction

B-cell depleting strategies based on anti-CD20 antibodies are widely used in B-cell hematologic malignancies and across a variety of autoimmune disorders [1,2]. They significantly reduce humoral immunity in the long term and have been shown to be associated with a higher risk of SARS-CoV-2 long infection and severity [3]. Although COVID-19 is widely diagnosed by a PCR test of nasopharyngeal swabs (NSs), there are pitfalls in such a diagnosis in B-cell-depleted patients with a COVID-19 infection. We herein report COVID-19 pneumonia diagnosed by bronchoalveolar lavage fluid (BALF) in two patients with a PCR-negative finding in NSs and no upper respiratory symptoms during lymphoma remission. Furthermore, their blood test with lymphopenia, BALF with decreased CD4/CD8 ratio, and increased neutralizing antibody titer suggested that CD4 T-cell responses are associated with more efficient viral clearance in immunocompromised patients.

## Case presentation

Case 1

A 54-year-old woman was referred to our hospital with pneumonia, which had persisted for three months. The patient had a fever and a non-productive cough but no episodes of shortness of breath or upper respiratory symptoms. Her medical history included hypertension. She had been diagnosed with follicular lymphoma one year previously. She had completed six courses of treatment for lymphoma with obinutuzumab and bendamustine nine months previously and remained in remission. She was taking sulfamethoxazole-trimethoprim and acyclovir to prevent pneumocystis pneumonia and herpes virus infections. She was vaccinated four times against SARS-CoV-2 and NS tests for SARS-CoV-2 and influenza virus antigens were negative. Based on the suspicion of bacterial pneumonia, the patient was treated with antibiotics but did not improve.

The patient’s vital signs were as follows: temperature 38.5°C, blood pressure 135/80 mmHg, heart rate 98 beats/min, respiratory rate 12 beats/min, and oxygen saturation (SpO _2_ ) 96% on room air. On auscultation, both lungs were clear. The patient was neurologically intact and had no skin lesions or enlarged lymph nodes. The other physical examination results were unremarkable.

**Figure 1:**
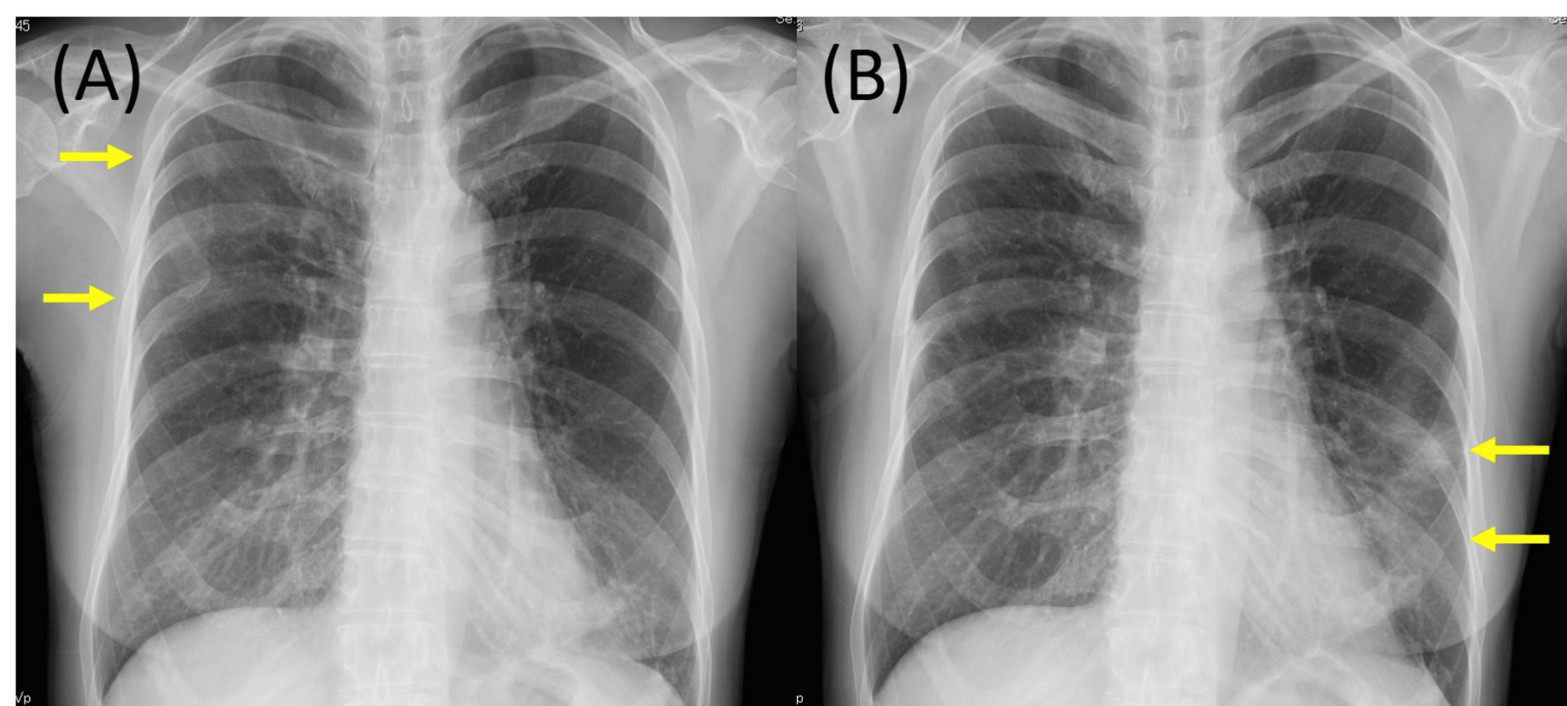
Chest X-ray in case 1 (A) Chest X-ray 1 month before the first consultation showing ground-glass opacities (GGOs) in the right upper lung. (B) Repeat chest X-ray at the first consultation showing improved previous opacities but a new GGO in the left lung.

**Figure 2:**
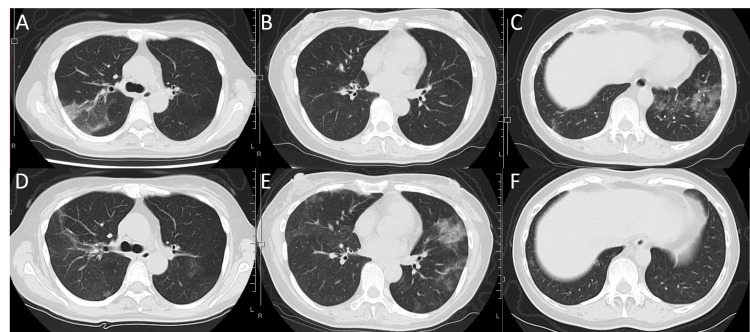
Chest CT in case 1 Chest CT one month before the first consultation showing ground-glass opacities (GGOs) in the right superior lobe on pleural side (A) and left inferior lobe(C) of the lung. The lingular lobe of the left lung is clear (B). Repeat chest CT at the first consultation showing improvement of opacities that were previously observed in the right superior lobe (D) and left inferior lobe (F) of the lung. New opacities were detected in the lingular and inferior lobes of the left lung (E).

The initial laboratory analysis revealed lymphocytopenia and tests for beta-D-glucan, cytomegalovirus antigenemia, and collagen-related autoantibodies were negative. SARS-Cov-2 PCR of the NS was negative, and she retained sufficient SARS-Cov-2 anti-spike IgG (Table 1). Chest radiography and CT showed new ground-glass opacities in the right middle, left inferior, and lingular lobes. The shadows that had previously been observed in the right superior and left inferior lobes disappeared (Figures 1, 2).

**Table 1:** Laboratory examination in case 1 at the first consultation.

Parameters	Laboratory values		Reference values	
White blood cells	2.5 × 10^3^ /mL		3.5-9.0 × 10^3^ /mL	
Lymphocytes	500 /μL		1500-3500 /μL	
Hemoglobin	9.1 g/dL		12-16 g/dL	
Platelets	127 × 10^3^ /mL		130-400 × 10^3^ /mL	
C-reactive protein	8.7 mg/dL		0-0.3 mg/dL	
Lactate dehydrogenase	263 U/L		119-229 U/L	
Kreb von den Lungen-6	259 U/L		<500 U/L	
Beta-D-glucan	5.7 pg/mL		<200 pg/mL	
Cytomegalovirus antigenemia	(-)		(-)	
SARS-Cov-2 anti-spike IgG	1129 U/mL		<0.8 U/mL	
SARS-Cov-2 anti-N protein IgG	(-)		(-)	

An analysis of the patient’s bronchoalveolar lavage (BAL) demonstrated lymphocytosis (51%) and a decreased CD4/CD8 ratio of 0.27. Bacterial and antimicrobial bronchoalveolar lavage fluid (BALF) cultures were negative. SARS-CoV2 PCR of the patient’s BALF was positive, and the cycle threshold was 27 (N2); therefore, the patient was diagnosed with COVID-19. The patient was three months past the onset of symptoms and did not have respiratory failure at diagnosis. Therefore, no antivirals or steroids were administered. At four months after the onset, fever and cough persisted, and a blood test showed an elevated CRP of 11 mg/dL. The patient was symptomatically treated with acetaminophen without respiratory failure. At 5 months after the onset, the symptoms improved, and CT showed that the pneumonia had disappeared.

Case 2

A 66-year-old man, who had been diagnosed with mantle lymphoma three years previously and had been treated with bendamustine, rituximab, and auto peripheral blood stem cell transplantation was found to still be in remission with rituximab. The patient was administered rituximab three months previously. He was referred to our hospital with a fever, non-productive cough and CT findings that showed pneumonia, which had persisted for one month. Initially, there appeared to be upper respiratory symptoms, but these had disappeared by the time of examination. Although he had never been vaccinated, he was treated with tixagevimab and cilgavimab one year previously to prevent the onset and severity of COVID-19. He was also taking sulfamethoxazole-trimethoprim to prevent pneumocystis pneumonia. Because a SARS-Cov-2 PCR finding of the NS was negative, the patient was treated with antibiotics for bacterial pneumonia, but his symptoms did not improve.

The patient’s vital signs were as follows: temperature 39.5°C, blood pressure 146/90 mmHg, heart rate 110 beats/min, respiratory rate 14 beats/min, and oxygen saturation (SpO_2_) 91% on room air. On auscultation, both lungs were clear. The patient had no skin lesions or enlarged lymph nodes. The other physical examination results were unremarkable.

**Figure 3:**
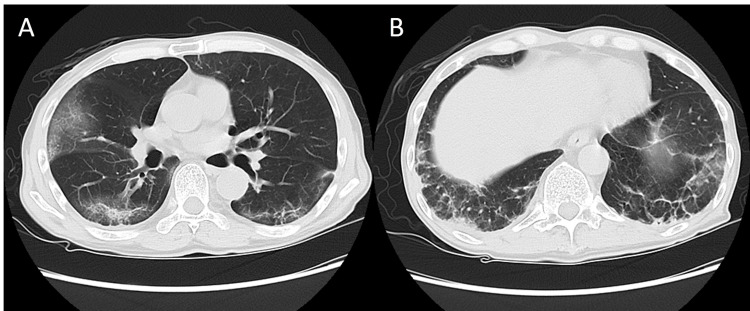
Chest CT in case 2 Chest CT showing GGO in the right middle lobe (A) and a linear shadow and GGO in the lateral and dorsal lower lobe (B).

The initial laboratory analysis revealed lymphocytopenia and tests for beta-D-glucan, cytomegalovirus antigenemia, and collagen-related autoantibodies were negative (Table 2). CT showed ground-glass opacities on the right middle and bilateral lower lobes on the pleural side (Figure 3).

**Table 2:** Laboratory investigation in case 2 at the first consultation.

Parameters	Laboratory values		Reference values	
White blood cells	2.5 × 10^3^ /mL		3.5-9.0 × 10^3^ /mL	
Lymphocytes	300 /μL		1500-3500 /μL	
Hemoglobin	9.2 g/dL		12-16 g/dL	
Platelets	264 × 10^3^ /mL		130-400 × 10^3^ /mL	
C-reactive protein	4.3 mg/dL		0-0.3 mg/dL	
Lactate dehydrogenase	240 U/L		119-229 U/L	
Kreb von den Lungen-6	816 U/L		<500 U/L	
Beta-D-glucan	9.0 pg/mL		<20 pg/mL	
Cytomegalovirus antigenemia	(-)		(-)	
Immunoglobulin G	425 mg/dL		870-1700 mg/dL	

An analysis of the patient’s BAL demonstrated lymphocytosis (87%) and a decreased CD4/CD8 ratio of 0.06. Bacterial and antimicrobial BALF cultures were negative. SARS-CoV2 PCR of the patient’s BALF was positive, and the cycle threshold was 29 (N2); therefore, the patient was finally diagnosed with COVID-19. Although the patient was one month past the onset of symptoms, he had mild respiratory failure. He was therefore given dexamethasone 6 mg once daily for 10 days, but his symptoms flared up after the end of the treatment. Symptomatic treatment was continued and his symptoms and shadow on CT improved at four months after the onset. 

## Discussion

There is a correlation between the detection of SARS-CoV2 in BALF and lymphocytosis in BALF in COVID-19-infected patients [4,5]. SARS-CoV2 PCR of BALF from both patients was positive, and the cycle thresholds were less than 30. Their CT showed ground-glass opacities predominantly on the pleural side, consistent with viral pneumonia. Various tests were negative for collagen disease and opportunistic and bacterial infections, and there was no history of suspected drug-induced pneumonia. They were therefore diagnosed with COVID-19 pneumonia.

In patients with B-cell depletion and prolonged COVID-19, SARS-CoV-2 NS specimens can be PCR-negative while BALF is PCR-positive. As there was a report that clinical recurrence occurred with a PCR-positive finding in a BALF specimen despite demonstrating early PCR-negative results in NS, in such cases, the nasopharyngeal viral load decreases rapidly from the early infection, but in the lower respiratory tract, it may remain higher for longer periods and also decrease more slowly [6,7]. SARS-CoV-2 uses angiotensin-converting enzyme 2 (ACE2) as a viral receptor to enter the human host cell, and ACE2 is especially highly expressed in lung alveolar epithelial cells and small intestinal epithelial cells [8]. In addition, ACE2 is abundantly expressed on the surface of alveolar type II pneumocytes, which remain targets of viral entry and replication, but it is also thought that maintaining normal ACE2 levels in the lung is beneficial to recover from inflammatory lung disease [9]. ACE2 orchestrates the metabolism of bradykinin in the lung, inhibiting effects such as vasodilation and increased vascular permeability, and also cleaves angiotensin II to angiotensin (1-7), which exerts vasodilatory, anti-inflammatory, and anti-fibrotic effects [8]. ACE2 upregulation contributes to the recovery of airway inflammation and lung injury but increases the opportunity for further viral cell replication and spread. This may be one reason why SARS-CoV-2 is present and detectable only in the lungs at the time of diagnosis in patients with delayed viral clearance. Due to low humoral responses, serum antibodies are not useful for an ancillary diagnosis or for determining a previous infection. Therefore, in B-cell-depleted patients with no upper respiratory symptoms and PCR-negative in NS but evidence of pneumonia on CT, the performance of early bronchoscopy with BAL is important for diagnosing or detecting the recurrence of COVID-19 and for ruling out opportunistic infections.

It has been reported that COVID-19 vaccination before rituximab therapy allows most patients with B-cell hematological malignancies to acquire and retain neutralizing antibodies [10]. The patient in case 1 was vaccinated four times before obinutuzumab treatment, so she retained adequate antibodies. Despite this, she had delayed virus clearance, which was thought to be due not only to low humoral immune responses but also to low cellular responses.

An immune response is important for viral clearance. However, a study on patients with complete absence of B-cells due to X-linked agammaglobulinemia who developed COVID-19 reported that they were able to recover from the infection [11]. This suggests that immune responses are not universally required for viral clearance or clinical recovery. In patients with B-cell depletion, the presence of a CD4 T-cell response is associated with more efficient viral clearance. It is thought that enhanced CD4 T-cell responses contribute to viral clearance in B-cell-depleted patients [12].

Obinutuzumab is an anti-CD20 monoclonal antibody that primarily affects B-cells. Bendamustine is an alkylating agent that causes prolonged lymphocytopenia, particularly CD4 T-cell depletion [13]. The median time from the end of obinutuzumab-bendamustine treatment for B-cell recovery has been reported to be 24 months, and the time to CD4 T-cell recovery is seven to nine months [14,15]. It has been reported that bendamustine use and multiple treatment lines for B-cell lymphoma were the significant independent predictors of prolonged viral shedding time [16]. In case 1, after treatment with obinutuzumab and bendamustine, the patient's lymphocyte count was <500 /µL. The patient in case 2 had also lymphopenic, 300 /µL. In general, the CD4/CD8 ratio of the BALF in patients with COVID-19 pneumonia was normal [4], but the BALF of these two patients showed a decreased CD4/CD8 ratio. These results suggest that cellular responses were persistently impaired, which is thought to be one of the causes of prolonged COVID-19 infection. Even if antibodies are adequate, we must be careful of prolonged COVID-19 infection owing to CD4 T-cell depletion and low humoral immune responses in patients treated with obinutuzumab or rituximab and bendamustine.

## Conclusions

In patients with prolonged COVID-19 pneumonia, NS may be PCR-negative for SARS-CoV-2. Even when NS specimens are PCR-negative without upper respiratory symptoms, early BAL for PCR should be performed in evidence of viral pneumonia on CT in immunocompromised patients. In particular, patients with low CD4 T-cell responses are at higher risk of persistent SARS-CoV-2 infection.
